# Tryptophan Metabolites as Biomarkers for Esophageal Cancer Susceptibility, Metastasis, and Prognosis

**DOI:** 10.3389/fonc.2022.800291

**Published:** 2022-02-28

**Authors:** Yun Chen, Jianliang Chen, Dainian Guo, Peixuan Yang, Shuang Chen, Chengkuan Zhao, Chengcheng Xu, Qiuzhen Zhang, Chaoxian Lin, Shilong Zhong, Shuyao Zhang

**Affiliations:** ^1^Department of Pharmacy, Guangzhou Red Cross Hospital, Jinan University, Guangzhou, China; ^2^Clinical Laboratory, Cancer Hospital of Shantou University Medical College, Shantou, China; ^3^Department of Pharmacy, Cancer Hospital of Shantou University Medical College, Shantou, China; ^4^Health Management Center, The First Affiliated Hospital of Shantou University Medical College, Shantou, China; ^5^Department of Pharmacology, Shantou University Medical College, Shantou, China; ^6^Department of Pharmacology, Shantou Chaonan Minsheng Hospital, Shantou, China; ^7^Department of Pharmacy, Guangdong Provincial People’s Hospital, Guangdong Academy of Medical Sciences, Guangzhou, China

**Keywords:** tryptophan metabolism, esophageal cancer, susceptibility, metastasis, prognosis, circulating biomarker

## Abstract

**Background:**

Perturbation of tryptophan (TRP) metabolism contributes to the immune escape of cancer; however, the explored TRP metabolites are limited, and their efficacy in clarifying the susceptibility and progression of esophageal cancer (EC) remains ambiguous. Our study sought to evaluate the effects of the TRP metabolic profile on the clinical outcomes of EC using a Chinese population cohort; and to develop a risk prediction model targeting TRP metabolism.

**Method:**

A total of 456 healthy individuals as control subjects and 393 patients with EC who were followed up for one year as case subjects were enrolled. Quantification of the plasma concentrations of TRP and its metabolites was performed using HPLC-MS/MS. The logistic regression model was applied to evaluate the effects of the clinical characteristics and plasma metabolites of the subjects on susceptibility and tumor metastasis events, whereas Cox regression analysis was performed to assess the overall survival (OS) of the patients.

**Results:**

Levels of creatinine and liver enzymes were substantially correlated with multiple metabolites/metabolite ratios in TRP metabolism, suggesting that hepatic and renal function would exert effects on TRP metabolism. Age- and sex-matched case–control subjects were selected using propensity score matching. Plasma exposure to 5-HT was found to be elevated 3.94-fold in case subjects (N = 166) compared to control subjects (N = 203), achieving an AUC of 0.811 for predicting susceptibility event. Subsequent correlation analysis indicated that a higher plasma exposure to 5-HIAA significantly increased the risk of lymph node metastasis (OR: 2.16, *p* = 0.0114). Furthermore, it was figured out that OS was significantly shorter for patients with elevated XA/KYN ratio (HR: 1.99, *p* = 0.0016), in which medium and high levels of XA/KYN versus low level had a significantly lower OS (HR: 0.48, *p* = 0.0080 and HR: 0.42, *p* = 0.0031, respectively).

**Conclusion:**

This study provides a pivotal basis for targeting endogenous TRP metabolism as a potential therapeutic intervention.

## 1 Introduction

Esophageal cancer (EC) refers to a malignant digestive tract cancer that develops from the aberrant proliferation of the esophageal squamous epithelium or glandular epithelium. Owing to its insidious onset and scarce early detection methods, EC is usually diagnosed as advanced or metastatic cancer, with a five-year survival rate of less than 16.8% (1). Metabolic reprogramming often occurs in the cancer microenvironment (2). Increasing evidence suggests that the rapid progression of cancer is due to the uncontrolled maintenance of immune homeostasis (3, 4), whereas the depletion of tryptophan (TRP) is a pivotal factor in cancer progression (5, 6). Immune homeostasis is susceptible to low extracellular TRP concentration, and the resultant TRP depletion leads to a proliferative block of T cells through the GCN2 pathway, thereby establishing the significant role of TRP metabolism in maintaining immune homeostasis (7).

Catalyzed by specific enzyme activities, TRP serves as a substrate for three different branches (i.e., kynurenine pathway (KP), 5-hydroxytryptamine (5-HT) pathway, and indole pathway) (8, 9), thereby giving rise to the formation of several molecules such as kynurenine (KYN), 5-HT, and 3-indolepropionic acid (IPA). KP metabolites, namely, kynurenic acid (KYNA), 3-hydroxykynurenine (3-HK), 3-hydroxyanthranilic acid (3-HAA), and xanthurenic acid (XA), might be subsequently released into the surroundings. While binding to aromatic receptors (AHRs), KP metabolites constitute the link between chronic inflammation and cancer progression, and further facilitate the decrease in immune surveillance (10, 11). Under the transformation of tryptophan hydroxylase, 5-HT and 5-hydroxyindoleacetic acid (5-HIAA) are generated, of which excessive upregulation of 5-HT could trigger overstimulation of growth function and accelerate cancer progression (5, 6). The remaining pathway mediated by the action of intestinal flora is the channel of IPA and 3-indoleacetic acid (IAA) formation, of which IPA is considered an effective free radical scavenger of indoleamine (12).

Given the rather complex network of TRP metabolic reactions, the regulation of vital immunosuppressive metabolites has become an attractive target for cancer therapy. Nevertheless, pathological shifts in the TRP metabolic profile have shown inconsistent trends in patients with different cancers. Previously, a nested case–control study was conducted which found that serum levels of 3-HAA and 3-HAA/3-HK were negatively correlated with the risk of pancreatic cancer, while KYN/TRP was not significantly correlated with the risk of pancreatic cancer (13). Another prospective trial revealed that the plasma levels of KYN, TRP, and KYN/TRP were significantly higher in healthy individuals than in patients with breast cancer (14). In addition, the only published study in regard to variation in the TRP metabolic profile in patients with EC merely incorporated a partial profile into a small-scale cohort (15). The association between circulating TRP and its metabolites and EC clinical outcomes has not been fully evaluated in a large-scale prospective study. Hence, current research has proven inconclusive in patients with EC, and more detailed characterization and quantification are urgently needed to systematically elucidate the extent to which the TRP metabolites can explain the EC disease state.

Herein, along the metabolite–clinical phenotype–study endpoint axis, we performed this study to investigate the impact of the TRP metabolic profile on the clinical primary outcomes of EC (i.e., events of susceptibility, lymph node metastasis, distant metastasis, and overall survival (OS) of patients) using a Chinese population cohort.

## 2 Materials and Methods

### 2.1 Study Design

A prospective study of the Chinese population was performed. First, clinical indexes were investigated in all subjects (456 healthy individuals and 393 patients with EC) to explain individual differences in the TRP metabolic profile. Later, the effects of plasma exposure to TRP and its metabolites on clinical endpoint events were assessed, namely, susceptibility, lymph node metastasis, distant metastasis, and OS.

### 2.2 Study Population

From August 2018 to November 2018, the study population, namely, 456 healthy individuals (control subjects) and 393 patients with histologically confirmed EC (case subjects), were sequentially recruited at the Cancer Hospital of Shantou University Medical College and the First Affiliated Hospital of Shantou University Medical College. Consistent baseline information was obtained for both groups from the hospital database, namely, demographics, blood routine examination, and biochemical measurements.

The exclusion criteria for the selected subjects were as follows: (1) age <18 years; (2) renal insufficiency (defined as serum creatinine [CREA] concentration >3 times the upper limit of normal [345 μmol/L], renal transplantation, or dialysis); (3) liver insufficiency (defined as serum transaminase concentrations >3 times the upper limit of normal [120 U/L] or a cirrhosis diagnosis); (4) pregnancy or lactation; (5) poor compliance or inability to complete the test; (6) patients who underwent anti-cancer treatments during this period; and (7) a history of other malignancies.

An additional exclusion criterion was required for control subjects: metabolic diseases such as diabetes, cancer, cardiovascular disease, severe infections, gout, and other end-stage diseases.

**Figure 1:**
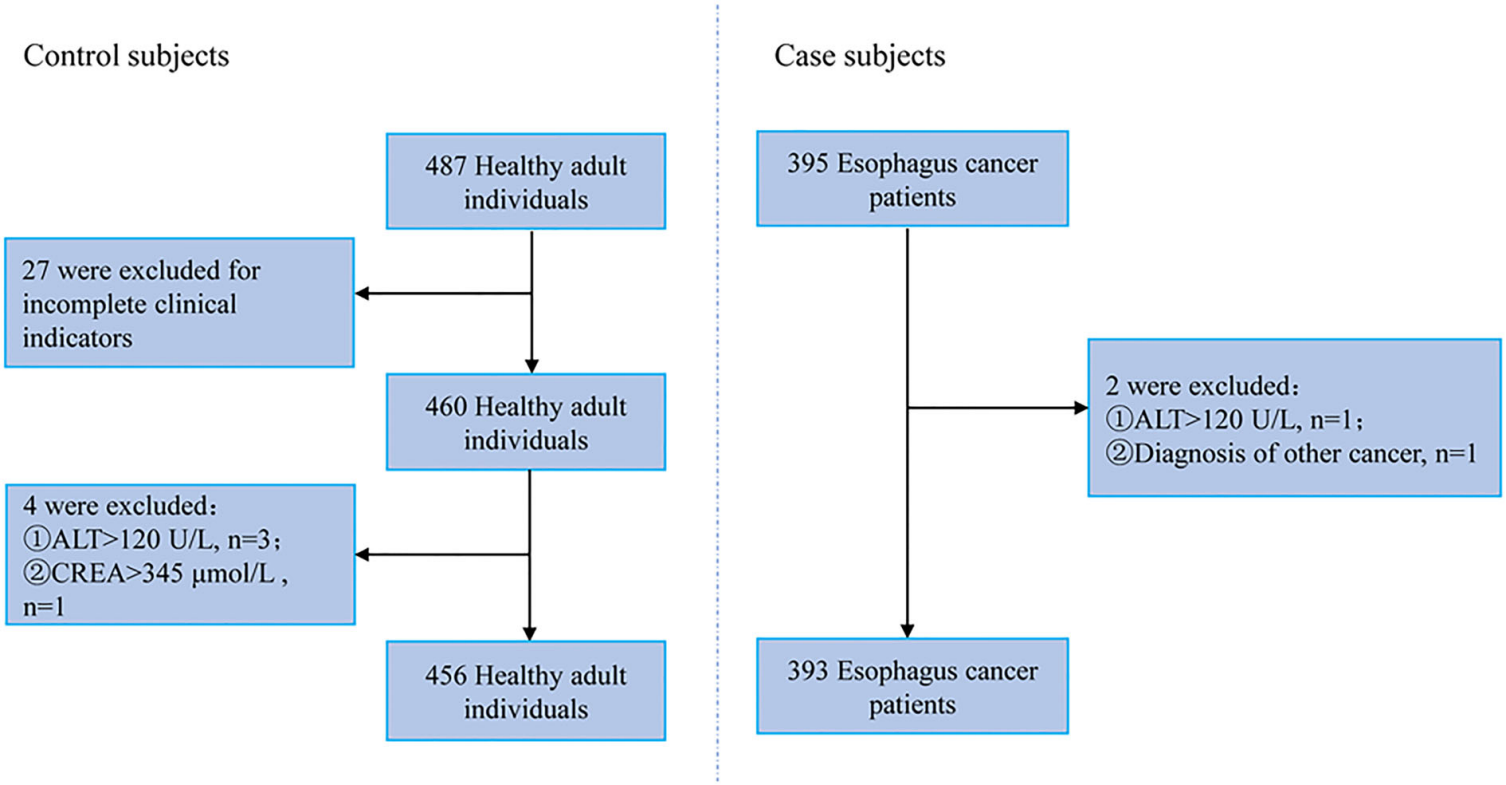
Workflow of sample selection.

The study was approved by the Ethics Committee of Guangzhou Red Cross Hospital (2020-109-02) and conducted in accordance with the Declaration of Helsinki principles. All subjects signed informed consent documentation. The workflow of sample selection is depicted in **Figure 1**.

### 2.3 Plasma Sample Preparation

Fasting blood collection in the early morning for both groups of subjects was required to minimize the influence of nutrition on the plasma levels of TRP metabolites. The patients pathologically diagnosed with EC did not receive radiotherapy or chemotherapy at this stage. Each blood sample was placed in EDTA anticoagulation tubes and centrifuged at 2,095×*g* for 10 min at 4°C within 2 h. Plasma and blood cells were separated and stored at −80°C for future analysis.

### 2.4 Clinical Endpoint

Clinical endpoints included susceptibility event, tumor metastasis event, and OS of patients.

A case–control study was conducted on susceptibility event. The initial sample population included 393 patients with EC and 456 healthy individuals. According to the propensity score matching (PSM) principle, 166 patients with EC and 203 healthy individuals were selected (*p >*0.05).

Tumor metastasis refers to the continued growth of cancer cells from the primary site (i.e., squamous or glandular epithelium of the esophagus) to other sites *via* lymphatic channels, blood vessels, or body cavities, including lymph node metastasis and distant metastasis. Lymph node metastasis refers to mediastina, neck, clavicle, axilla, abdomen, and peritoneum lymph node metastasis. Distant metastasis refers to metastasis to the lung, liver, bone, and other sites. The evaluation of tumor metastasis was mainly based on the clinical diagnosis, tumor staging, pathology, and imaging examination (abdominal and pelvic CT or MRI and chest X-ray) of patients at the Cancer Hospital of Shantou University Medical College.

OS was defined as the time from the start of randomization to the death of any cause and was mainly evaluated based on in-hospital outpatient follow-up and out-of-hospital telephone follow-up. Follow-up information for each patient was obtained from the hospital database and telephone interviews. The telephone follow-up period was as follows: advanced-stage patients were followed-up every 3 months, and early stage patients were followed-up every 6 months. The last follow-up period of this study was December 2019, and the patients were followed up for 1 year.

### 2.5 Quantification of Plasma Concentrations of TRP and Their Metabolites

A sensitive high-performance liquid chromatography-tandem mass spectrometry (HPLC–MS/MS) assay was established for the simultaneous quantification of TRP, KYN, KYNA, 3-HAA, XA, 5-HT, 5-HIAA, IPA, and IAA in human plasma. Analysis was performed using an HPLC system (LC-20A, Shimadzu) coupled with an API 4000 triple-quadrupole mass spectrometer (AB, Sciex).

Nine analytes and internal standards (TRP-*d5* and KYN-*d4*) were isolated from human plasma by liquid–liquid extraction with prechilled acetonitrile and then separated on an Acquity XSelect HSS T3 column (2.1 mm × 100 mm, 3.5 μm) at a flow rate of 0.30 ml/min by the gradient of the mobile phase consisting of 0.01% (*v*/*v*) formic acid in water (A) and acetonitrile (B). The following gradient program was used: 0–0.1 min 5% B; 0.1–0.5 min 5→60% B; 0.5–2.8 min 60% B; 2.8–2.9 min 60→5% B; 2.9–5.0 min 5% B.

Mass detection was performed using an API 4000 triple-quadrupole mass spectrometer under the positive electrospray ionization mode. Electrospray voltage (IS) was set at 5,500 V, desolvation gas temperature was set at 550°C, and ion source gas 1 (CS1) and ion source gas 2 (CS2) were set at 50 psi. The air curtain gas (CUR) was 25 psi. Declustering potential (DP) and collision energy (CE) were optimized for each analyte and internal standard. Ion transitions and optimized multiple reaction monitoring (MRM) parameters are shown in **Table S1**.

### 2.6 Data Preprocessing and Analysis

Demographic and clinical characteristics were summarized using counts (percentages) for categorical variables and medians (interquartile ranges [IQR]) for continuous variables. Considering that the concentration ranges of all metabolites were skewed, logarithmic transformation was performed prior to analysis. Spearman correlation analysis was applied to study the association between plasma concentrations of upstream and downstream metabolites (i.e., TRP with all downstream metabolites, KYN with KP downstream metabolites and 5-HT with 5-HIAA). If *p <*0.05, metabolite ratios were also included in the list of dependent variables.

Linear regression analysis was performed to assess the effects of baseline demographic and clinical characteristics on the plasma metabolites/metabolite ratios. Factors with *p <*0.05 after univariate linear regression were employed into the multivariate regression model, in which *p <*0.05 was considered as the independent factor of metabolites/metabolite ratios. The R^2^ was used to evaluate the interpretability of the model.

Based on the propensity index, the appropriate PSM method (“MatchIt” package) was employed to correct the imbalance of baseline data between the study population of case subjects and control subjects for inclusion of the population in the susceptibility event study calculated using the nearest neighbor method. Age and sex were used as covariates to condense a comprehensive score, with a paired caliper value of 0.02. A logistic regression model was applied to evaluate the effects of clinical characteristics and plasma metabolites of the subjects on susceptibility and tumor metastasis events, and Cox regression analysis was performed to assess the OS of patients, while the odds ratio (OR), hazard ratio (HR), and 95% confidence interval (95%CI) were calculated. Variables with *p <*0.05 were entered into the multivariate model, and only variables with *p <*0.05 considered as independent impact factors, were retained in the model. Furthermore, prognostic models of clinical events were constructed for each predictive variable by receiver-operating characteristic (ROC) curves using the area under the curve (AUC) to measure the diagnostic effectiveness. The optimal cutoffs were calculated by selecting the data point that maximized the true-positive rate and minimized the false-positive rate. The Kaplan–Meier method was conducted to assess the effects of every independent variable associated with OS, in which patients were further stratified into three groups: low, moderate, and high in terms of the quartiles of their plasma levels, and the *p*-value was analyzed with the log-rank test.

**Figure 2:**
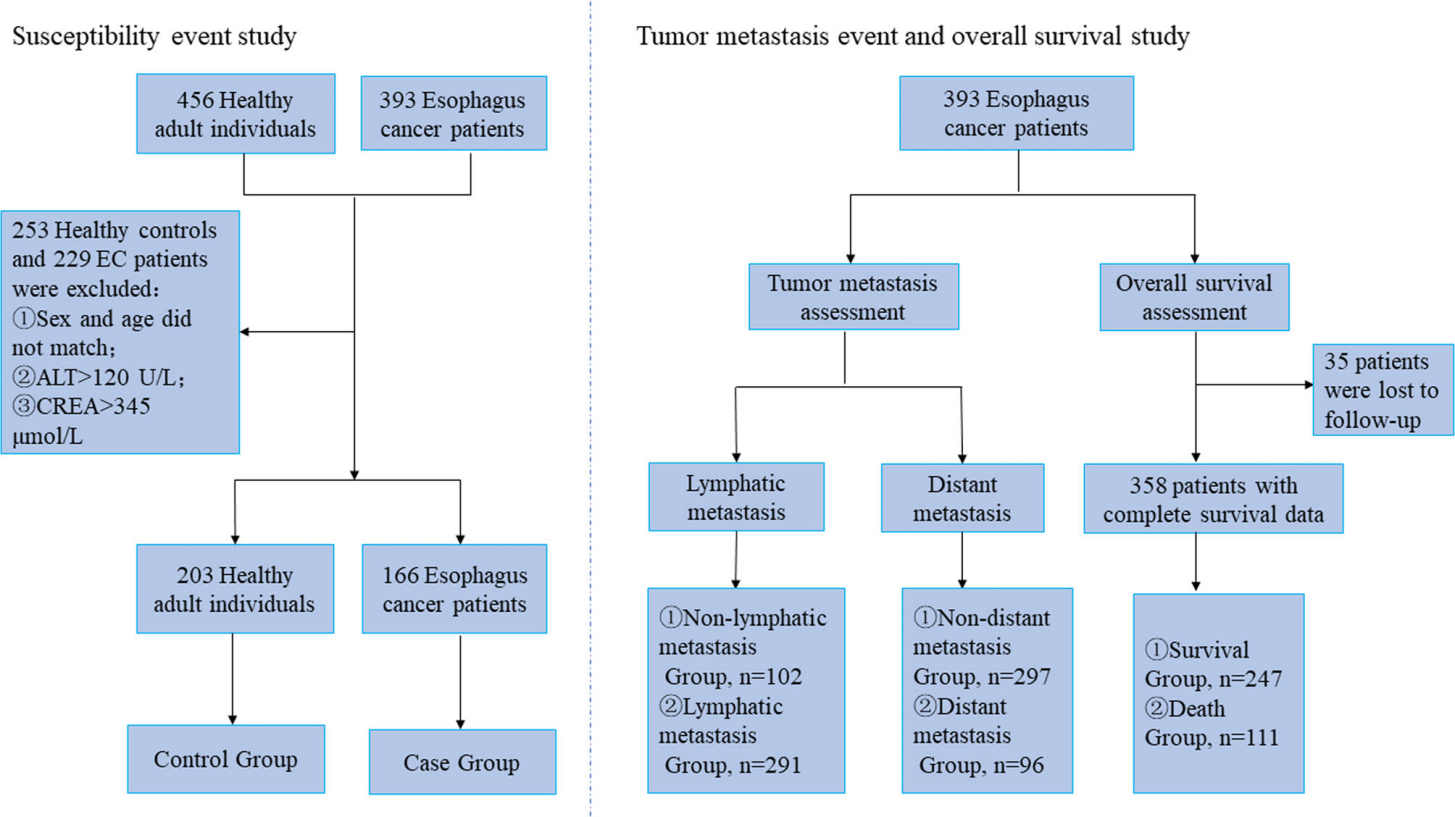
Flow chart of experiment design and sample selection.

The criterion for statistical significance was set at *p <*0.05. All data were analyzed using SAS 9.4 (SAS Institute, Cary, NC, USA), R (version 3.2.3, http://www.R-project.org/), and GraphPad Prism 6. A flowchart of the experimental design and sample selection process is depicted in **Figure 2**.

## 3 Results

### 3.1 General Characteristics and Metabolite-to-Metabolite Correlation Analysis of the Participants

Based on these criteria, we ultimately included 456 healthy individuals and 393 patients with EC. The demographic characteristics and clinical data of the enrolled subjects are shown in **Tables S2**, **S3**. Of the 849 participants eligible for this study, 456 healthy individuals were assigned to the control cohort, and 393 patients with EC were assigned to the case cohort. In the metabolite-to-metabolite correlation analysis (**Table S4**), all subjects revealed that the concentration of TRP was significantly and positively associated with the concentrations of five metabolites (KYN, KYNA, XA, 3-HAA, and IAA), and KYN was positively correlated with KYNA, XA, and 3-HAA (r >0, *p <*0.05), whereas a significant negative correlation was found between TRP and IPA in control subjects (r <0, *p <*0.05). Therefore, the corresponding ratios were included in the list of dependent variables for further correlation analyses.

### 3.2 Effects of Baseline Characteristics on the Plasma Exposure of TRP and Its Metabolites in Case Subjects

Univariate linear regression analysis revealed the following: (1) high CREA levels were associated with the plasma levels of KYN, KYNA, 5-HIAA, IPA, and IAA; (2) coagulation index of patients, namely, platelet (PLT), was correlated with plasma exposure to multiple metabolites, namely, TRP, KYNA, 5-HT, and IPA; (3) male patients showed higher plasma exposure to TRP, XA, and 3-HAA; (4) other clinical indexes for hepatic function [i.e., aspartate aminotransferase (AST)], nutrition [i.e., albumin (ALB), globulin (GLB), and total protein (TPROT)], coagulation (i.e., PLT), and immune [i.e., red blood cell (RBC) and hemoglobin (HGB)] exhibited certain effects on plasma exposure of metabolites (**Table S2**).

Similarly, univariate linear regression analysis of the baseline characteristics of plasma metabolite ratios showed that low ALB levels significantly affected multiple metabolite ratios, namely, KYN/TRP, KYNA/TRP, XA/TRP, 3-HAA/TRP, and 3-HAA/KYN ratios. Second, age, CREA level, and RBC count were the secondary impact factors influencing the plasma levels of KYN/TRP, KYNA/TRP, XA/TRP, 3-HAA/TRP, XA/KYN, and 3-HAA/KYN. Other indexes that characterized hepatic function (i.e., AST), nutrition (i.e., TPROT), and cardiac function [i.e., lactate dehydrogenase (LDH)] exhibited certain effects on plasma exposure to metabolites (**Table S2**).

Based on the results of the univariate linear regression analysis, significant factors serving as covariates were incorporated into the multivariate linear regression model of each metabolite/metabolite ratio. Among these variables, it was found that the CREA level in the case subjects independently affected several metabolites/metabolite ratios of the TRP metabolic profile, namely, KYN, KYNA, 5-HIAA, IPA, KYN/TRP, KYNA/TRP, XA/KYN, and 3-HAA/KYN. The AST level was considered the secondary factor, correlating with the plasma levels of KYN, 5-HIAA, IPA, KYN/TRP, KYNA/KYN, and XA/KYN. It was speculated that the hepatic and renal functions pf the patients might be involved in the dynamic changes in the TRP metabolic profile. Corresponding interpretations of the TRP metabolic profile to the clinical baseline are presented in **Table S2**. Of note, platelet count was considered significantly associated with plasma exposure to 5-HT, which was consistent with a previous report published in Blood (16), demonstrating the reliability of the present experimental results.

### 3.3 Effects of Baseline Characteristics on the Plasma Exposure of TRP and Its Metabolites in Control Subjects

Univariate linear regression analysis showed that plasma exposure to multiple metabolites was significantly influenced by the levels of ALB, GLB, ALB/GLB, RBC, and HGB, associated metabolites, namely, TRP, KYN, KYNA, XA, 3-HAA, 5-HIAA, and IPA. Second, except for 3-HAA, plasma exposure to TRP and its metabolites could be significantly affected by two demographic indexes (i.e., sex and age). Hepatic and renal function indexes (alanine aminotransferase [ALT], AST, and CREA) and neutrophil ratio (NE) were correlated with plasma exposure to TRP, KYN, KYNA, XA, 3-HAA, 5-HIAA, and IAA. The effects of the remaining factors on the metabolites in the control subjects are shown in **Table S3**.

Similarly, univariate linear regression analysis showed that age significantly affected the metabolite ratios in the control subjects except for 3-HAA/TRP. In accordance with the above results, the plasma levels of multiple metabolite ratios were still significantly affected by sex and the levels of ALB, GLB, and ALB/GLB; the effects of the remaining factors on the metabolite ratios are shown in **Table S3**.

These factors were incorporated into a multivariate linear regression model of individual metabolites/metabolite ratios. Multivariate linear regression identified that the CREA levels in the control subjects independently affected several metabolites/metabolite ratios in the TRP metabolic profile, namely, IAA, KYN/TRP, KYNA/TRP, IAA/TRP, and 3-HAA/KYN. Hepatic function indexes (i.e., alkaline phosphatase [ALP] and ALT) were considered as secondary factors involving metabolites/metabolite ratios, namely, TRP, KYN, KYNA, 3-HAA, 5-HT, KYN/TRP, and KYNA/KYN. Consistent with the case cohort, hepatic and renal function were similarly involved in the dynamics of the TRP metabolic profile in control subjects, and platelets in the control cohort were similarly independent influencers of plasma exposure to 5-HT. According to previous studies, platelets and mast cells were proposed to be the vital reservoirs of 5-HT, and secretion of 5-HT would lead to increased uptake of 5-HT by circulating platelets and mast cells (17, 18).

### 3.4 Contribution of Plasma Exposure of TRP and Its Metabolites to Susceptibility, Metastasis, and OS

#### 3.4.1 Results of the PSM

Given the significant differences in age and sex in the original two cohorts, confounding bias could alter the veracity of the findings on cancer susceptibility event; therefore, matching the two cohorts was required to increase the statistical power of the present case–control study.

The MatchIt package of R was used to match the age and sex of the case and control subjects. Before matching, the age of the control subjects was 48.00 (37.50, 56.00) years, accounting for 53.73% of males and 46.27% of females, while the age of case subjects was 63.00 (58.00, 67.00) years, accounting for 77.35% of males and 22.65% of females. The PSM scores of the control and case subjects were 0.28 ± 0.27 and 0.67 ± 0.27, respectively.

**Figure 3:**
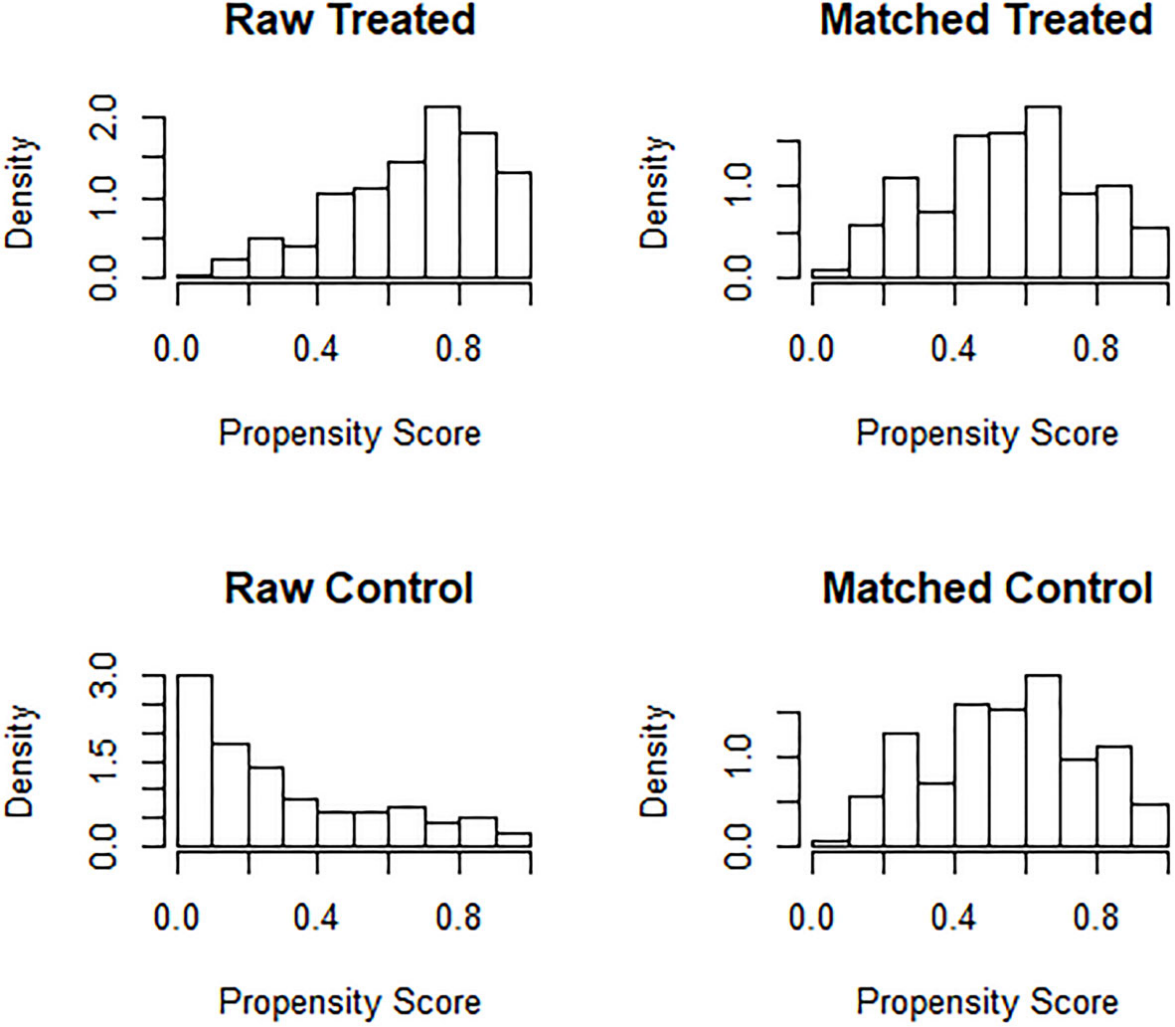
Histogram of propensity scores of two groups before and after matching.

*Via* the nearest neighbor method of PSM, the age of control subjects was 57.00 (52.00, 62.00) years old, of which 63.55% were male and 36.45% were female, while the age of case subjects was 58.00 (53.00, 63.00) years old, of which 68.67% were male, and 31.33% were female. There was no statistically significant difference between the two groups (*p >*0.05). The PSM scores of the two groups were 0.55 ± 0.22 in control subjects and 0.55 ± 0.22 in case subjects, which proved that the age and sex of both groups were balanced and comparable (**Figure 3**). After matching, the two cohorts were subjected to a subsequent case-control statistical study, as detailed in **Table S5** and **Figure 2**.

#### 3.4.2 Effects of TRP and Its Metabolites on Susceptibility Event of the EC and Their Predictive Value

Subjects with susceptibility events included 166 age- and sex-matched patients with EC and 203 healthy individuals. Univariate logistic regression showed that plasma exposure to TRP and its eight metabolites, six metabolite ratios, NE, RBC, HGB, PLT, ALT, AST, glutamyl transferase (GGT), and ten other biochemical indicators were associated with susceptibility to EC (**Table 1**). Furthermore, the significantly related factors were included in the multivariate logistic regression model. It was found that only high levels of 5-HT and ALP and low levels of HGB were independent factors influencing susceptibility to EC. Compared with the control group, the plasma exposure to 5-HT in the case group increased 3.94 times, ALP level increased 1.58 times, and HGB level decreased 1.28 times. In the whole multivariate regression model, the concordant part accounted for 99.5% and the discordant part accounted for 0.5%, indicating that the prediction accuracy of the whole regression model was high. The results of this section imply that a high level of 5-HT incorporating ALP and a low HGB level would increase the susceptibility to EC.

**Table 1:** Associations of metabolites and baseline data with susceptibility event.

Characteristics		Control subjects	Case subjects	Univariable Analysis		Multivariable Analysis	
		N (%) or Median (IQR)	N (%) or Median (IQR)	OR (95%CI)	*p*	OR(95%CI)	*p*
**Demographic data**							
Total number		203	166				
Age, years		57.00 (52.00, 62.00)	58.0 (53.00, 63.00)	1.02 (1.00–1.05)	0.0905		
Sex	Female	74 (36.45)	52 (31.33)	1.02 (1.00–1.94)	0.3018		
	Male	129 (63.55)	114 (68.67)				
**Blood routine examination**							
WBC, 10^9^/L		6.54 (5.67, 7.62)	5.85 (4.20, 8.07)	0.95 (0.88–1.03)	0.2498		
NE, %		54.00 (48.64, 60.00)	66.71 (56.26, 75.00)	1.10 (1.08–1.13)	<.0001		
RBC, 10^12^/L		4.81 (4.54, 5.16)	4.21 (3.68, 4.69)	0.15 (0.09–0.24)	<.0001		
HGB, g/L		151.00 (141.00, 161.00)	118.95 (105.20, 133.20)	0.89 (0.87–0.91)	<.0001	0.83 (0.75–0.91)	0.0002
PLT,10^9^/L		215.00 (186.00, 246.00)	228.00 (172.00, 283.90)	1.00 (1.00–1.00)	0.0119		
**Biochemical measurements**							
ALT, U/L		24.00 (20.00, 32.00)	20.00 (14.00, 28.00)	0.97 (0.96–0.99)	0.0010		
AST, U/L		28.00 (25.00, 33.00)	19.00 (16.00, 23.00)	0.84 (0.81–0.88)	<.0001		
CREA, μmol/L		79.00 (66.00, 89.00)	76.00 (67.00, 87.00)	1.00 (0.99–1.01)	0.4065		
GGT, U/L		30.00 (21.00, 45.00)	20.00 (16.00, 32.00)	0.99 (0.99–1.00)	0.0211		
ALP, U/L		78.50 (64.00, 94.00)	126.00 (104.00, 140.00)	1.06 (1.04–1.07)	<.0001	1.07 (1.03–1.12)	0.0003
LDH, U/L		178.00 (163.00, 207.00)	152.00 (135.00, 177.00)	0.99 (0.98–0.99)	0.0001		
TPROT, g/L		75.00 (72.10, 77.90)	67.80 (62.60, 71.70)	0.78 (0.75–0.83)	<.0001		
ALB, g/L		45.50 (43.40, 46.70)	39.60 (35.90, 42.70)	0.57 (0.50–0.64)	<.0001		
GLB, g/L		29.50 (26.80, 32.30)	28.05 (24.70, 30.60)	0.91 (0.86–0.95)	0.0001		
ALB/GLB		1.52 (1.38, 1.69)	1.44 (1.23, 1.61)	0.16 (0.06–0.38)	<.0001		
GLU, mmol/L		5.56 (5.24, 6.23)	5.02 (4.60, 5.45)	0.55 (0.43–0.71)	<.0001		
**Plasma concentrations and Plasma ratios**							
TRP, ng/ml		10,900.00 (9,600.00, 12,300.00)	8,235.00 (6,960.00, 9,790.00)	0.01 (0.01–0.04)	<.0001		
KYN, ng/ml		323.00 (272.00, 382.00)	260.00 (216.00, 345.00)	0.14 (0.07–0.29)	<.0001		
KYNA, ng/ml		8.33 (6.76, 10.20)	6.42 (5.05, 8.09)	0.11 (0.06–0.22)	<.0001		
XA, ng/ml		9.11 (7.17, 11.00)	6.59 (5.17, 8.13)	0.10 (0.05–0.20)	<.0001		
3-HAA, ng/ml		0.92 (0.71, 1.15)	1.10 (0.80, 1.55)	2.50 (1.41–4.42)	0.0017		
5-HT, ng/ml		6.89 (4.90, 9.86)	16.60 (9.70, 28.60)	5.57 (3.71–8.36)	<.0001	98.47 (8.00–∞)	0.0003
5-HIAA, ng/ml		10.30 (7.44, 16.30)	9.13 (7.48, 14.80)	0.77 (0.48–1.25)	0.2902		
IPA, ng/ml		90.30 (56.40, 172.00)	53.20 (31.00, 101.00)	0.64 (0.51–0.80)	0.0001		
IAA, ng/ml		183.00 (137.00, 239.00)	152.00 (105.00, 244.00)	0.60 (0.41–0.88)	0.0087		
KYN/TRP		2.91E−02 (2.50E−02, 3.45E−02)	3.27E−02 (2.64E−02, 4.23E−02)	2.97 (1.50–5.89)	0.0018		
KYNA/TRP		7.40E−04 (6.20E−04, 9.18E−04)	7.35E−04 (6.33E−04, 8.86E−04)	1.07 (0.57–2.02)	0.8400		
XA/TRP		8.40E−04 (6.59E−04, 1.01E−03)	8.13E−04 (5.97E−04, 1.04E−03)	0.66 (0.35–1.21)	0.1777		
3-HAA/TRP		8.47E−05 (6.30E−05, 1.13E–04)	1.35E−02 (9.07E−05, 1.84E−04)	6.52 (3.59–11.86)	<.0001		
IPA/TRP		8.15E−03 (4.82E−03, 1.62E−02)	6.59E−03 (3.51E−03, 1.36E−02)	0.86 (0.69–1.06)	0.1511		
IAA/TRP		1.70E−02 (1.29E−02, 2.32E−02)	1.94E−02 (1.35E−02, 2.75E−02)	1.64 (1.12–2.40)	0.0116		
KYNA/KYN		2.55E−02 (2.20E−02, 3.02E−02)	2.33E−02 (1.88E−02, 2.85E−02)	0.32 (0.16–0.67)	0.0024		
XA/KYN		2.78E−02 (2.18E−02, 3.36E−02)	2.42E−02 (1.86E−02, 3.17E−02)	0.43 (0.26–0.72)	0.0012		
3-HAA/KYN		2.80E−03 (2.13E−03, 3.54E−03)	3.78E−03 (2.79E−03, 5.53E−03)	4.46 (2.50–7.97)	<.0001		

Variables with p <0.05 were entered into the multivariable model, and only variables with p <0.05 were retained in the model.

WBC, white blood cell; NE, neutrophil ratio; RBC, red blood cell; HGB, hemoglobin; PLT, platelet; ALT, alanine aminotransferase; AST, aspartate aminotransferase; CREA, creatinine; GGT, glutamyl transferase; ALP, alkaline phosphatase; LDH, lactate dehydrogenase; TPROT, total protein; ALB, albumin; GLB, globulin; GLU, glucose; TRP, tryptophan; KYN, kynurenine; KYNA, kynurenic acid; XA, xanthurenic acid; 3-HAA, 3-hydroxyanthranilic acid; 5-HT, 5-hydroxytryptamine; 5-HIAA, 5-hydroxyindoleacetic acid; IPA, 3-indolepropionic acid; IAA, 3-indoleacetic acid; IQR, interquartile range; OR, odd ratio; CI, confidence interval.

**Figure 4:**
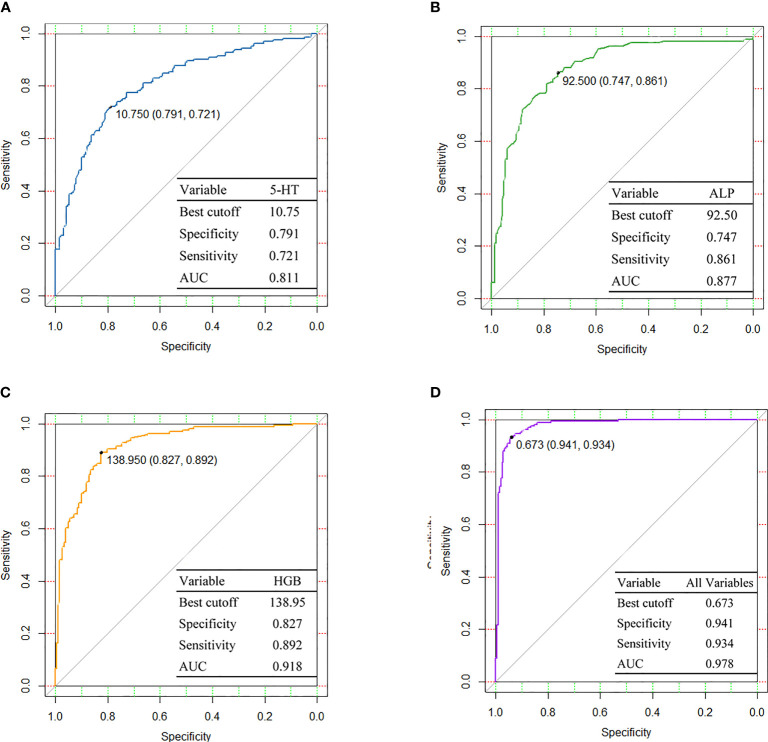
Diagnostic performances are shown by ROC curves among **(A)** 5-HT, **(B)** ALP, **(C)** HGB, and **(D)** All variables. 5-HT, 5-hydroxytryptamine; ALP, alkaline phosphatase; HGB, hemoglobin; AUC, area under curve.

Subsequently, we constructed ROC curves with the variables constituted by three independent factors and three merging factors individually to obtain the predictive efficacy for susceptibility events, as shown in **Figure 4**. ROC analysis of 5-HT for predicting susceptibility events achieved an AUC of 0.811, with the best cutoff of 10.75 ng/ml, sensitivity of 0.721, and specificity of 0.791. After integrating three independent factors, 5-HT, ALP, and HGB, the predictive effectiveness of the combined variables was increased substantially to 0.978.

#### 3.4.3 Effects of TRP and Its Metabolites on EC Metastasis

In this subsection, we discuss lymph node and distant metastases. A total of 291 patients with lymph node metastasis and 102 patients without lymph node metastasis were included in the analysis of lymph node metastasis events, and the results of univariate logistic regression analysis showed that 5-HT, 5-HIAA, and TPROT were significantly associated with lymph node metastasis (**Table 2**). Further significant correlation factors were included in the multivariate logistic regression model, and only high plasma exposure to 5-HIAA remained an independent factor for lymph node metastasis. Plasma exposure to 5-HIAA exhibited a 1.24-fold increase in the lymph node metastasis subjects compared to non-lymph node metastasis subjects. The consistent and inconsistent parts of the multivariate regression model accounted for 61.4 and 38.0%, respectively. In contrast, 96 patients with distant metastasis and 297 patients with non-distant metastasis were included, and univariate logistic regression analysis revealed that the plasma levels of IPA and XA/KYN, age, several blood routine indexes [i.e., white blood cell (WBC) and NE], and several biochemical indexes (i.e., AST, GGT, and ALP) of patients were correlated with distant metastasis (**Table 3**). However, multivariate logistic regression analysis revealed that only low WBC and high GGT levels were independent factors for distant metastatic event, whereas metabolites exerted no significant effect on distant metastatic event.

**Table 2:** Associations of metabolites and baseline data with lymph node metastasis event.

Characteristics		Non-lymphatic metastasis Group	Lymphatic metastasis Group	Univariable Analysis		Multivariable Analysis	
		N (%) or Median (IQR)	N (%) or Median (IQR)	OR (95%CI)	*p*	OR (95%CI)	*p*
**Demographic data**							
Total number		102	291				
Age, years		64.00 (59.00, 68.00)	63.0 (57.00, 67.00)	0.98 (0.95–1.01)	0.1471		
Sex	Female	27 (26.47)	62 (21.31)	1.33 (0.79–2.24)	0.2844		
	Male	75 (73.53)	229 (78.69)				
**Medical history**							
Hypertension	No	81 (79.41)	230 (79.04)	1.02 (0.59–1.79)	0.9365		
	Yes	21 (20.59)	61 (20.96)				
Diabetes	No	95 (93.14)	262 (90.03)	1.50 (0.64–3.54)	0.3530		
	Yes	7 (6.86)	29 (9.97)				
Cerebral Infarction	No	98 (96.08)	284 (97.59)	0.60 (0.17–2.11)	0.4286		
	Yes	4 (3.92)	7 (2.41)				
Family History of Cancer	No	97 (95.10)	279 (95.88)	0.83 (0.29–2.43)	0.7398		
	Yes	5 (4.90)	12 (4.12)				
**Blood routine examination**							
WBC, 10^9^/L		6.54 (5.67, 7.62)	5.47 (4.20, 7.74)	0.96 (0.91–1.02)	0.1766		
NE, %		65.26 (53.70, 75.92)	67.02 (56.91, 74.97)	1.00 (0.99–1.02)	0.6830		
RBC, 10^12^/L		4.20 (3.57, 4.65)	4.14 (3.60, 4.61)	0.94 (0.68–1.30)	0.6946		
HGB, g/L		122.20 (108.80, 135.60)	119.20 (108.30, 132.50)	0.99 (0.98–1.00)	0.1880		
PLT, 10^9^/L		233.00 (178.00, 288.00)	223.00 (168.00, 281.70)	1.00 (1.00–1.00)	0.2223		
**Biochemical measurements**							
ALT, U/L		19.00 (13.00, 26.00)	19.00 (14.00, 28.00)	1.01 (0.99–1.02)	0.4163		
AST, U/L		18.00 (15.00, 22.00)	19.00 (15.00, 24.00)	1.03 (1.00–1.06)	0.0893		
CREA, μmol/L		79.00 (66.00, 92.00)	78.00 (68.00, 88.00)	1.00 (0.99-1.01)	0.6656		
GGT, U/L		20.00 (16.00, 29.00)	21.00 (16.00, 32.00)	1.00 (0.99–1.01)	0.7067		
ALP, U/L		120.00 (103.00, 135.00)	121.00 (104.00, 142.00)	1.00 (1.00–1.00)	0.9988		
LDH, U/L		145.00 (129.00, 168.00)	153.00 (133.00, 181.00)	1.00 (1.00–1.01)	0.1395		
TPROT, g/L		69.90 (61.80, 74.50)	67.40 (61.80, 71.30)	0.96 (0.94–0.99)	0.0132		
ALB, g/L		40.00 (36.90, 42.70)	39.20 (35.50, 41.90)	0.95 (0.90–1.00)	0.0514		
GLB, g/L		28.40 (24.60, 31.90)	27.70 (24.70, 30.70)	0.97 (0.93–1.02)	0.2661		
ALB/GLB		1.42 (1.27, 1.56)	1.42 (1.23, 1.58)	0.87 (0.34–2.19)	0.7646		
GLU, mmol/L		5.18 (4.71, 5.75)	5.03 (4.65, 5.54)	0.97 (0.82–1.15)	0.6966		
**Living habit**							
Smoking	No	43 (42.16)	114 (39.31)	1.13 (0.71–1.78)	0.6139		
	Yes	59 (57.84)	176 (60.69)				
Drinking	No	71 (69.61)	194 (66.90)	1.13 (0.70–1.85)	0.6149		
	Yes	31 (30.39)	96 (33.10)				
**Plasma concentrations**							
TRP, ng/ml		8,725.00 (6,960.00, 10,000.00)	8,250.00 (6,980.00, 9,580.00)	0.83 (0.42–1.65)	0.5988		
KYN, ng/ml		261.50 (214.00, 339.00)	275.00 (224.00, 350.00)	1.21 (0.64–2.29)	0.5565		
KYNA, ng/ml		6.56 (5.15, 7.94)	6.49 (5.29, 8.09)	1.31 (0.71–2.42)	0.3916		
XA, ng/ml		6.54 (5.08, 7.98)	6.65 (5.06, 8.60)	1.07 (0.67–1.71)	0.7727		
3-HAA, ng/ml		1.09 (0.78, 1.48)	1.13 (0.82, 1.54)	1.07 (0.62–1.85)	0.8203		
5-HT, ng/ml		12.90 (8.55, 23.50)	16.40 (9.50, 28.40)	1.33 (1.03–1.72)	0.0303		
5-HIAA, ng/ml		8.04 (6.72, 13.40)	11.00 (8.01, 16.80)	2.18 (1.21–3.97)	0.0095	2.16 (1.19–3.93)	0.0114
IPA, ng/ml		53.30 (28.40, 145.00)	52.60 (28.40, 98.60)	0.93 (0.74–1.17)	0.5280		
IAA, ng/ml		155.00 (115.00, 256.00)	159.00 (107.00, 254.00)	1.02 (0.73–1.41)	0.9156		
**Plasma ratios**							
KYN/TRP		3.22E−02 (2.73E−02, 3.75E−02)	3.34E−02 (2.79E−02, 4.39E−02)	1.42 (0.75–2.69)	0.2795		
KYNA/TRP		7.68E−04 (6.31E−04, 9.05E−04)	7.91E−04 (6.48E−04, 9.38E−04)	1.61 (0.75–3.44)	0.2201		
XA/TRP		7.94E−04 (5.85E−04, 9.98E−04)	8.13E−03 (6.00E−04, 1.09E−03)	1.25 (0.70–2.22)	0.4549		
3-HAA/TRP		1.28E−04 (9.00E−05, 1.85E−04)	1.39E−04 (9.87E−05, 1.89E−04)	1.06 (0.65–1.74)	0.8209		
IAA/TRP		1.89E−02 (1.46E−02, 2.80E−02)	2.02E−02 (1.32E−02, 3.24E−02)	1.06 (0.77–1.45)	0.7272		
KYNA/KYN		2.47E−02 (1.91E−02, 2.91E−02)	2.29E−02 (1.86E−02, 2.86E−02)	1.02 (0.53–1.99)	0.9454		
XA/KYN		2.38E−02 (1.80E−02, 3.22E−02)	2.38E−02 (1.75E−02, 3.29E−02)	0.96 (0.62–1.51)	0.8706		
3-HAA/KYN		3.11E−03 (1.34E−03, 4.83E−03)	3.09E−03 (0.00, 4.73E−03)	0.91 (0.55–1.52)	0.7246		

Variables with p <0.05 were entered into the multivariable model, and only variables with p <0.05 were retained in the model.

Abbreviations as in **Table 1**.

**Table 3:** Associations of metabolites and baseline data with distant metastasis event.

Characteristics		Non-distant metastasis Group	Distant metastasis Group	Univariable Analysis		Multivariable Analysis	
		N (%) or Median (IQR)	N (%) or Median (IQR)	OR (95%CI)	*p*	OR (95%CI)	*p*
**Demographic data**							
Total number		297	96				
Age, years		64.00 (59.00, 68.00)	60.00 (56.00, 66.00)	0.96 (0.93–0.99)	0.0047		
Sex	Female	64 (21.55)	25 (26.04)	0.78 (0.46–1.33)	0.3612		
	Male	233 (78.45)	71 (73.96)				
**Medical history**							
Hypertension	No	230 (77.44)	81 (84.38)	0.63 (0.34–1.18)	0.1488		
	Yes	67 (22.56)	15 (15.63)				
Diabetes	No	268 (90.24)	89 (92.71)	0.73 (0.31–1.72)	0.4671		
	Yes	29 (9.76)	7 (7.29)				
Cerebral Infarction	No	288 (96.97)	94 (97.92)	0.68 (0.15–3.21)	0.6272		
	Yes	9 (3.03)	2 (2.08)				
Family History of Cancer	No	281 (94.61)	95 (98.96)	0.19 (0.02–1.41)	0.1037		
	Yes	16 (5.39)	1 (1.04)				
**Blood routine examination**							
WBC, 10^9^/L		5.98 (4.30, 8.23)	5.00 (3.80, 8.23)	0.90 (0.83–0.97)	0.0090	0.91 (0.83–0.99)	0.0292
NE, %		67.50 (57.07, 75.91)	65.70 (53.10, 73.90)	0.98 (0.97–1.00)	0.0148		
RBC, 10^12^/L		4.20 (3.60, 4.62)	4.14 (3.68, 4.62)	0.87 (0.63–1.20)	0.3926		
HGB, g/L		120.80 (109.60, 133.60)	118.50 (105.50, 131.60)	1.00 (0.99–1.01)	0.4068		
PLT, 10^9^/L		232.40 (170.65, 287.00)	209.50 (169.00, 275.00)	1.00 (1.00–1.00)	0.2516		
**Biochemical measurements**							
ALT, U/L		18.00 (14.00, 26.00)	20.00 (14.00, 30.00)	1.02 (1.00–1.03)	0.0785		
AST, U/L		18.00 (15.00, 23.00)	19.00 (16.00, 25.00)	1.03 (1.01–1.06)	0.0081		
CREA, μmol/L		79.00 (68.00, 90.00)	74.00 (66.00, 86.00)	0.99 (0.97–1.00)	0.0896		
GGT, U/L		21.00 (16.00, 30.00)	22.00 (17.00, 39.00)	1.01 (1.00–1.02)	0.0024	1.02 (1.01–1.03)	0.0001
ALP, U/L		117.50 (103.00, 138.50)	126.00 (104.00, 148.00)	1.01 (1.00–1.01)	0.0231		
LDH, U/L		151.00 (133.00, 175.00)	158.00 (126.00, 197.00)	1.00 (1.00–1.00)	0.3640		
TPROT, g/L		67.60 (61.60, 72.10)	67.40 (62.10, 71.50)	1.00 (0.97–1.03)	0.9952		
ALB, g/L		39.55 (35.85, 42.20)	39.20 (35.30, 42.10)	0.99 (0.94–1.04)	0.5556		
GLB, g/L		27.65 (24.60, 30.95)	28.20 (25.10, 31.10)	1.01 (0.97–1.06)	0.6247		
ALB/GLB		1.42 (1.25, 1.58)	1.40 (1.22, 1.60)	0.77 (0.30–1.96)	0.5789		
GLU, mmol/L		5.09 (4.65, 5.64)	5.07 (4.68, 5.68)	1.06 (0.89–1.25)	0.5339		
**Living habit**							
Smoking	No	114 (38.51)	43 (44.79)	0.77 (0.49–1.23)	0.2759		
	Yes	182 (61.49)	53 (55.21)				
Drinking	No	202 (68.24)	63 (65.63)	1.13 (0.69–1.83)	0.6339		
	Yes	94 (31.76)	33 (34.38)				
**Plasma concentrations**							
TRP, ng/ml		8,180.00 (6,910.00, 9,650.00)	8,390.00 (7,040.00, 9,580.00)	1.31 (0.65–2.65)	0.4494		
KYN, ng/ml		262.00 (222.00, 339.00)	286.00 (229.00, 375.00)	1.83 (0.94–3.59)	0.0776		
KYNA, ng/ml		6.39 (5.23, 7.92)	6.62 (5.32, 8.52)	1.51 (0.81–2.83)	0.1982		
XA, ng/ml		6.62 (5.09, 8.51)	6.51 (4.68, 8.41)	0.82 (0.51–1.31)	0.4079		
3-HAA, ng/ml		1.10 (0.78, 1.56)	1.21 (0.88, 1.49)	1.11 (0.65–1.91)	0.7003		
5-HT, ng/ml		15.55 (9.26, 28.00)	15.50 (9.25, 26.95)	1.03 (0.80–1.31)	0.8399		
5-HIAA, ng/ml		10.55 (7.48, 16.80)	10.60 (7.85, 16.20)	0.83 (0.47–1.48)	0.5282		
IPA, ng/ml		50.60 (25.60, 95.80)	55.65 (32.55, 118.50)	1.28 (1.02–1.61)	0.0330		
IAA, ng/ml		158.00 (112.00, 257.00)	152.00 (102.00, 226.00)	0.83 (0.59–1.17)	0.2890		
**Plasma ratios**							
KYN/TRP		3.27E−02 (2.76E−02, 4.09E−02)	3.30E−02 (2.78E−02, 4.74E−02)	1.42 (0.74–2.73)	0.2984		
KYNA/TRP		7.82E−04 (6.38E−04, 9.37E−04)	8.07E−04 (6.72E−04, 9.66E−04)	1.15 (0.56–2.37)	0.7117		
XA/TRP		8.37E−04 (5.98E−04, 1.08E−03)	7.83E−04 (5.96E−04, 1.01E−03)	0.62 (0.34–1.11)	0.1064		
3-HAA/TRP		1.35E−04 (9.35E−05, 1.95E−04)	1.46E−04 (1.03E−04, 1.82E−04)	0.99 (0.60–1.62)	0.9629		
IAA/TRP		2.07E−02 (1.34E−02, 3.10E−06)	1.73E−02 (1.32E−02, 2.87E−02)	0.79 (0.57–1.11)	0.1704		
KYNA/KYN		2.38E−02 (1.90E−02, 2.92E−02)	2.36E−02 (1.76E−02, 2.79E−02)	0.81 (0.41–1.61)	0.5469		
XA/KYN		2.47E−02 (1.83E−02, 3.38E−02)	2.05E−02 (1.63E−02, 2.88E−02)	0.63 (0.40–0.99)	0.0460		
3-HAA/KYN		3.01E−03 (0.00, 4.86E−03)	3.25E−03 (2.18E−03, 4.66E−03)	0.76 (0.45–1.29)	0.3064		

Variables with p <0.05 were entered into the multivariable model, and only variables with p <0.05 were retained in the model.

Abbreviations as in **Table 1**.

#### 3.4.4 Effects of TRP and Its Metabolites on the OS of Patients With EC

**Figure 5:**
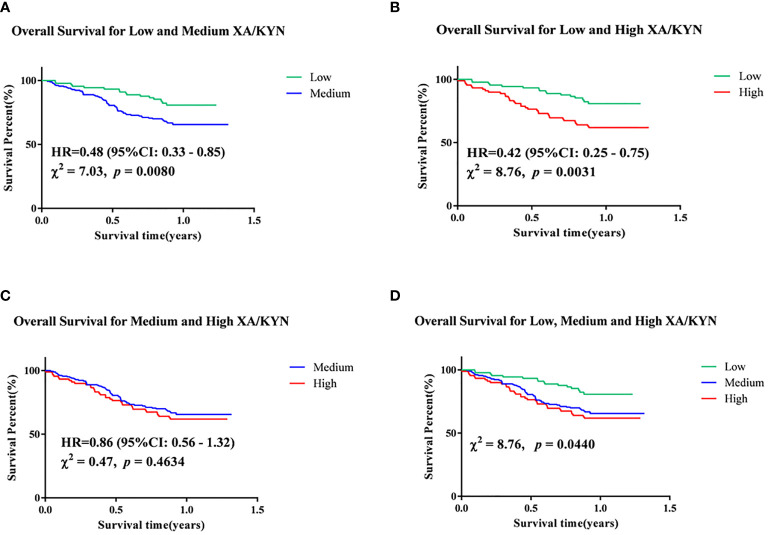
Kapan-Meier analyses of **(A)** low and medium XA/KYN, **(B)** low and high XA/KYN, **(C)** medium and high XA/KYN, and **(D)** low, medium and high XA/KYN for overall survival of patients. XA, xanthurenic acid; KYN, kynurenine.

Follow-up for this study was completed in December 2019, with survival data available for 358 patients. Of the enrolled patients with EC, 111 died, 247 survived, and 35 were lost to follow-up. Univariate Cox regression analysis showed that plasma levels of XA/TRP, 3-HAA/TRP, XA/KYN, and 3-HAA/KYN, male sex, comorbid hypertension, several blood routine indexes, and several biochemical indexes were significantly associated with the OS of patients (**Table 4**). Furthermore, significantly associated factors were included in the multivariate Cox regression model, and it was found that high levels of XA/KYN, NE, and low ALB levels independently and significantly affected the OS of patients. To clarify the risk of this event by XA/KYN, we further stratified the patients by quartiles of plasma XA/KYN levels and divided the patients into three groups: high, medium, and low. The Kaplan–Meier method was applied to analyze the differences in survival curves among different groups, as shown in **Figure 5**. Survival prognosis revealed that patients with a low plasma level of XA/KYN showed a significantly lower OS than patients with a medium plasma level of XA/KYN (HR: 0.48, *p* = 0.0080). A similar trend was observed between patients with low and high plasma levels of XA/KYN (HR: 0.42, *p* = 0.0031). However, there was no significant difference between patients with medium and high plasma XA/KYN levels (HR: 0.86, *p* = 0.4634), as depicted in **Figure 5**.

**Table 4:** Associations of metabolites and baseline data with overall survival of patients.

Characteristics		Survival Group	Death Group	Univariable Analysis		Multivariable Analysis	
		N (%) or Median (IQR)	N (%) or Median (IQR)	HR (95%CI)	*p*	HR (95%CI)	*p*
**Demographic data**							
Total number		247	111				
Age, years		63.00 (58.00, 67.00)	63.00 (59.00, 70.00)	1.02 (0.99–1.04)	0.1745		
Sex	Female	65 (26.32)	15 (13.51)	2.03 (1.18–3.50)	0.0109		
	Male	182 (73.68)	96 (86.49)				
**Medical history**							
Hypertension	No	203 (82.19)	81 (72.97)	1.61 (1.06–2.45)	0.0255		
	Yes	44 (17.81)	30 (27.03)				
Diabetes	No	228 (92.31)	102 (91.89)	1.01 (0.51–2.00)	0.9753		
	Yes	19 (7.69)	9 (8.11)				
Cerebral Infarction	No	242 (97.98)	106 (95.50)	1.93 (0.79–4.74)	0.1499		
	Yes	5 (2.02)	5 (4.50)				
Family History of Cancer	No	236 (95.55)	107 (96.40)	0.96 (0.35–2.60)	0.9339		
	Yes	11 (4.45)	4 (3.60)				
Stage	Early stage	52 (21.49)	7 (6.60)	3.30 (1.53–7.10)	0.0023		
	Advanced stage	190 (78.51)	99 (93.40)				
**Blood routine examination**							
WBC, 10^9^/L		5.50 (4.20, 7.74)	5.78 (4.06, 8.80)	1.05 (1.00–1.10)	0.0512		
NE, %		65.00 54.20, 72.64)	71.45 (62.61, 79.05)	1.03 (1.02–1.04)	<.0001	1.02 (1.00–1.03)	0.0240
RBC, 10^12^/L		4.30 (3.78, 4.67)	3.89 (3.47, 4.31)	0.55 (0.43–0.71)	<.0001		
HGB, g/L		123.40 (111.80, 135.30)	114.75 (101.90, 128.40)	0.99 (0.98–0.99)	0.0001		
PLT, 10^9^/L		220.00 (169.00, 279.20)	234.50 (174.00, 304.00)	1.00 (1.00–1.00)	0.0210		
**Biochemical measurements**							
ALT, U/L		20.00 (14.00, 28.00)	16.00 (12.00, 27.00)	0.98 (0.96–1.00)	0.0282		
AST, U/L		19.00 (16.00, 24.00)	17.00 (13.00, 23.00)	0.99 (0.97–1.01)	0.4240		
CREA, μmol/L		79.00 (70.00, 89.00)	76.00 (67.00, 90.00)	1.00 (0.98–1.01)	0.4003		
GGT, U/L		20.00 (16.00, 30.00)	22.50 (16.00, 39.00)	1.00 (1.00–1.01)	0.0111		
ALP, U/L		118.00 (101.00, 135.00)	123.00 (106.00, 145.00)	1.00 (1.00–1.01)	0.1912		
LDH, U/L		153.00 (133.00, 177.00)	150.50 (130.00, 174.00)	1.00 (1.00–1.00)	0.0171		
TPROT, g/L		69.20 (63.90, 73.00)	64.25 (58.30, 69.20)	0.97 (0.95–0.98)	<.0001		
ALB, g/L		40.50 (37.50, 42.70)	36.65 (33.50, 40.00)	0.87 (0.84–0.90)	<.0001	0.86 (0.82–0.90)	<.0001
GLB, g/L		28.20 (25.10, 31.00)	27.40 (23.60, 31.10)	0.97 (0.94–1.01)	0.1875		
ALB/GLB		1.42 (1.29, 1.58)	1.31 (1.18, 1.57)	0.30 (0.13–0.68)	0.0038		
GLU, mmol/L		5.04 (4.67, 5.49)	5.14 (4.63, 5.67)	1.09 (0.96–1.25)	0.1701		
**Living habit**							
Smoking	No	103 (41.87)	43 (38.74)	1.10 (0.75–1.61)	0.6327		
	Yes	143 (58.13)	68 (61.26)				
Drinking	No	170 (69.11)	71 (63.96)	1.18 (0.80–1.73)	0.4099		
	Yes	76 (30.89)	40 (36.04)				
**Plasma concentrations**							
TRP, ng/ml		8,550.00 (6,990.00, 9,920.00)	7,980.00 (6,740.00, 9,240.00)	1.00 (0.58–1.73)	0.9907		
KYN, ng/ml		274.00 (229.00, 349.00)	274.00 (229.00, 349.00)	0.78 (0.46–1.33)	0.3637		
KYNA, ng/ml		6.54 (5.33, 7.96)	6.54 (5.33, 7.96)	1.13 (0.66–1.94)	0.6512		
XA, ng/ml		6.49 (4.63, 8.35)	6.49 (4.63, 8.35)	1.73 (1.14–2.63)	0.0107		
3-HAA, ng/ml		1.09 (0.78, 1.48)	1.09 (0.78, 1.48)	1.48 (0.97–2.26)	0.0691		
5-HT, ng/ml		16.60 (9.07, 29.00)	16.60 (9.07, 29.00)	0.94 (0.77–1.15)	0.5514		
5-HIAA, ng/ml		9.89 (7.24, 16.30)	9.89 (7.24, 16.30)	1.29 (0.85–1.96)	0.2348		
IPA, ng/ml		52.80 (26.60, 109.00)	52.80 (26.60, 109.00)	1.15(0.95–1.38)	0.1529		
IAA, ng/ml		159.00 (108.00, 263.00)	159.00 (108.00, 263.00)	0.95 (0.73–1.24)	0.6856		
**Plasma ratios**							
KYN/TRP		3.33E−02 (2.76E−02, 4.23E−02)	3.26E−02 (2.76E−02, 4.41E−02)	0.77 (0.44–1.34)	0.3460		
KYNA/TRP		7.84E−04 (6.46E−04, 9.09E−04)	7.76E−04 (6.35E−04, 9.77E−04)	1.33 (0.74–2.40)	0.3429		
XA/TRP		7.54E−04 (5.73E−04, 1.02E−03)	8.96E−04 (6.94E−04, 1.13E−03)	2.20 (1.34–3.61)	0.0019		
3-HAA/TRP		1.30E−04 (9.48E−05, 1.77E−04)	1.55E−04 (1.01E−04, 2.11E−04)	1.47 (1.01–2.12)	0.0427		
IAA/TRP		1.97E−02 (1.34E−02, 3.30E−02)	2.11E−02 (1.36E−02, 2.80E−02)	0.95 (0.74–1.23)	0.6955		
KYNA/KYN		2.28E−02 (1.89E−02, 2.85E−02)	2.43E−02 (1.86E−02, 2.94E−02)	1.74 (0.96–3.15)	0.0668		
XA/KYN		2.32E−02 (1.63E−02, 3.03E−02)	2.70E-02 (1.96E−02, 3.53E−02)	1.95 (1.29–2.93)	0.0015	1.99 (1.30–3.04)	0.0016
3-HAA/KYN		3.00E−03 (0.00, 4.55E−03)	3.45E−03 (1.78E−03, 5.68E−03)	1.72 (1.16–2.55)	0.0073		

Variables with p <0.05 were entered into the multivariable model, and only variables with p <0.05 were retained in the model.

Abbreviations as in **Table 1**.

## Discussion

Reprogrammed TRP metabolism has been proven to directly cause carcinogenesis and cancer progression as an adaptive mechanism for tumors to escape immune surveillance and metastasize, rendering it critical for tight control to maintain healthy homeostasis (19). However, given the relatively complex profile of TRP metabolism, a simple one-branch model could not capture the complex dynamics of the multi-branched TRP metabolic network, whereas evidence to elucidate the dynamic shifts of tryptophan metabolite concentrations involved in the different disease states of EC was sparse. To the best of our knowledge, this work is the first systematic assessment of clinical endpoint events of EC between TRP metabolic profiles and clinical phenotypes, namely, susceptibility event, lymph node metastasis event, distant metastasis event, and OS. A comprehensive mathematical model was established to provide a predictive tool that could facilitate identification of potential pathological changes in TRP metabolism.

Existing reports suggest that hepatic and renal functions assumed the biological roles in the metabolic network of TRP, as our results indicated (20–22). Similarly, this study found that clinical hepatic and renal function indexes were tightly correlated with TRP metabolites. In addition to the reported KP metabolites, corresponding increases in IPA and 5-HIAA levels were also found in patients with EC with liver and kidney insufficiency. Under normal conditions, the TDO enzyme controls TRP flux in the liver and its availability in plasma, and may supply KYN to the extrahepatic pathway (21). Not to be stated, hepatic function exerted its prominent regulatory effect on the metabolic network of TRP. In an association study of renal function, Yilmaz et al. suggested that the ratio of TRP to KYN could reflect the oxidative stress status in chronic kidney disease patients undergoing hemodialysis and peritoneal dialysis (20). Numerous studies have also shown that TRP metabolism is associated with the severity of chronic kidney disease. Early reports showed that renal insufficiency would lead to decreased TRP levels and KYN accumulation in rats and humans. Moreover, a targeted metabolomic profiling of plasma exposure to TRP in terms of the diagnostic value of acute kidney injury was carried out in renal transplant patients, reaching a decision that low plasma exposure to TRP and high levels of KYN and KYN/TRP were powerful predictors of kidney injury (22). Consistent with previous studies, our data suggest that hepatic and renal function might directly contribute to TRP metabolism.

Additionally, after adjusting for several potential confounding variables, we evaluated whether the metabolic pathways and associated metabolites were altered in the different disease states of the EC. The principal findings were as follows: (1) TRP metabolism was significantly imbalanced in patients with EC. Three major metabolic pathways were severely dysregulated in both groups, in which the population with high plasma exposure to 5-HT significantly increased the risk of EC; (2) 5-HIAA was independently associated with increased lymph node metastasis risk; and (3) high plasma levels of XA/KYN conferred a risk effect on the occurrence of death, since KYN metabolism towards XA was significantly increased in the high-risk group of death events. The above results characterized their roles in carcinogenesis and potency as risk markers for EC metastasis and prognosis.

Recently, both *in vivo* and *in vitro* studies have shown that 5-HT serves as an effective mitogen in various cell types (23). Its specific receptor subtypes are associated with the progression of solid cancers and can affect the growth of various cancers (24). Accumulating evidence has shown that the level of platelet-derived 5-HT in the cancer microenvironment is higher than that in healthy individuals (25). Moreover, platelet aggregation is frequent in the environment of cancer thrombosis, resulting in the release of a large amount of 5-HT, which is one of the mechanisms of cancer progression and angiogenesis. Our results are consistent with these results. Univariate analysis demonstrated that the PLT level in patients with EC was significantly higher than that in healthy individuals and was significantly associated with plasma exposure to 5-HT in both groups (**Tables S2** and **S3**). Previous studies have shown that 5-HT executes physiological functions in the gastrointestinal tract, participates in esophageal and gastrointestinal motility, and is a mediator of the brain-gut connection (26). Additionally, 5-HT1, 5-HT2, 5-HT3, and 5-HT4 receptors perform local and systemic functions in human esophageal motility and the transient contraction of the esophageal sphincter (27). Among them, the 5-HT3 receptor also participates in visceral sensitization of esophageal sensitivity (28). Another study by Hempfling et al. showed that the esophagus had a wealth of 5-HT positive internal nerves, in which 5-HT could regulate the vagus nerve movement of the esophageal striated muscle (29), which suggests that 5-HT serves as an essential biomolecule in the regulation of the physiological state of the esophagus. Consistent with previous studies, our results suggested that 5-HT plays a crucial role in EC susceptibility and may be a potential biomarker for EC.

Regarding 5-HIAA, it has been recognized as a biomarker of neuroendocrine cancer and carcinoid syndrome, since the digestive tract is one of the primary sources of 5-HT and its metabolites (such as 5-HIAA), and the presence of non-endocrine malignant cells in the digestive tract might lead to an increase in these metabolites in body fluid and urine (1). To date, carcinoid cancer has been the only cancer diagnosed explicitly by measuring the urinary level of 5-HIAA in suspected patients (30). Nonetheless, as an effective marker of neuroendocrine cancer, there are limited data to support the prognostic role of 5-HIAA due to its differential expression and secretion. Insufficient evidence was observed for 5-HIAA as a prognostic marker owing to its limitation of high specificity yet low sensitivity (31). Van et al. indicated that the urinary level of 5-HIAA was an independent prognostic factor for the survival of patients with midgut cancers, whereas cumulative urinary 5‐HIAA could not predict the survival rate in multivariate analysis (32).

Other studies have also pointed out that urinary 5‐HIAA is an effective index of survival and prognosis in univariate analysis, except for multivariate analysis (33, 34). In our correlation study between metabolites and prognosis of patients, 5-HIAA was not found to be associated with OS (HR: 1.29, 95%CI: 0.85–1.96, *p* = 0.2348), thus revealing that this metabolite could not serve as a biomarker to aid in determining the prognosis of patients with EC yielded insufficient evidence. Previously, Cheng et al. found that the serum 5-HIAA/TRP level in EC patients with metastasis was higher than that non-metastatic patients, which is consistent with our results (15). In this study, we compared the TRP metabolic profile between patients with and without lymph node metastasis. Univariate analysis showed that 5-HT and 5-HIAA levels were increased in patients with lymph node metastasis, and high plasma exposure to 5-HIAA was the only independent factor influencing lymph node metastasis. Based on the above results, we speculated that plasma exposure to 5‐HIAA can be used as an effective biomarker to assist in diagnosing the risk of lymph node metastasis in patients with EC.

Interestingly, after exploring the association between metabolites and clinical endpoint events, this study suggested that the plasma level of XA/KYN significantly affected OS (HR: 1.99, 95%CI: 1.30–3.04, *p* = 0.0016), speculating that the metabolism of KYN in the direction of XA increased the risk of death. Moreover, it is significant to point out that this metabolite ratio was the first discovery of an association between the TRP metabolic profile and the prognosis of cancer patients.

The high enzyme activity of KP has been proposed to trigger anti-inflammatory and immunosuppressive mechanisms, thereby executing pathological functions such as activation of cancer and aromatic hydrocarbon receptor (AHR). Remarkably, activation of AHR drives the transformation of naive CD4^+^ cells to inhibit the Treg phenotype by reducing inflammatory cytokines and upregulating cytokines, thus promoting the occurrence and growth of malignant cancers (35). In addition, KYN, KYNA, and XA are all inducers of AHR activity, which could drive AHR activity and promote cancer cell migration (19). Furthermore, XA has been recognized as a regulator of glutamate synaptic transmission and a promising candidate as a peripheral biomarker of schizophrenia; however, the detailed mechanisms of cancer development have not been reported (36, 37). Additionally, it was worth noting that several prospective population-based studies had suggested that deficiency of vitamin B6 was associated with the increased risk of colorectal cancer and lung cancer (38–40). Several scientists have demonstrated that this association may be related to the inflammatory state, angiogenesis, DNA methylation, cell-mediated immune response, and other mechanisms (41). Moreover, if the body is deficient in vitamin B6, KYN metabolism would shift from NAD+ formation to XA and KYNA production (42). Hence, we envisioned the possibility that XA/KYN might affect the survival and prognosis of patients by affecting the concentration of vitamin B6; however, this hypothesis requires further examination.

However, a limitation of this study is that the sample size was relatively small. Nonetheless, we conducted a strict screening and quality control process on the original sample population, and conducted sample screening and correlation discussions for different clinical endpoint events. Most of our findings are in line with those of previous studies, proving the reliability of our results. There is no denying that a more extensive study cohort would be more convincing to clarify the association with clinical endpoint events. In the future, broader and more comprehensive inclusion of the study population would address this limitation.

## Conclusion

A comprehensive evaluation of the clinical predictive value of tryptophan metabolism in carcinogenesis and its potential as biomarkers for metastasis and prognosis of EC emerged from the present study. Our results demonstrated that elevated levels of 5-HT, 5-HIAA, and XA/KYN were observed in EC and might be part of the mechanism underlying the susceptibility, lymph node metastasis, and poor prognosis of EC, respectively. This study provides an essential theoretical basis for targeting endogenous TRP metabolism as a potential therapeutic intervention.

## Data Availability Statement

The original contributions presented in the study are included in the article/**Supplementary Material**. Further inquiries can be directed to the corresponding authors.

## Ethics Statement

The studies involving human participants were reviewed and approved by the Guangzhou Red Cross Hospital. The patients/participants provided their written informed consent to participate in this study.

## Author Contributions

YC, JC, DG, and PY performed the experiment, performed data analysis, and wrote the manuscript. SC participated in the experiment. CZ, CX, and QZ participated in patient recruitment. CL revised the manuscript; ShiZ and ShoZ designed the study and revised manuscript. All authors contributed to the article and approved the submitted version.

## Funding

This study was funded by the 2020 Guangdong Science and Technology Special Fund (“Large Special + Task List”) item (No: 200113165875501).

## Conflict of Interest

The authors declare that the research was conducted in the absence of any commercial or financial relationships that could be construed as a potential conflict of interest.

## Publisher’s Note

All claims expressed in this article are solely those of the authors and do not necessarily represent those of their affiliated organizations, or those of the publisher, the editors and the reviewers. Any product that may be evaluated in this article, or claim that may be made by its manufacturer, is not guaranteed or endorsed by the publisher.
